# Early ficolin-1 is a sensitive prognostic marker for functional outcome in ischemic stroke

**DOI:** 10.1186/s12974-016-0481-2

**Published:** 2016-01-20

**Authors:** R. Zangari, E. R. Zanier, G. Torgano, A. Bersano, S. Beretta, E. Beghi, B. Casolla, N. Checcarelli, S. Lanfranconi, A. Maino, C. Mandelli, G. Micieli, F. Orzi, E. Picetti, M. Silvestrini, N. Stocchetti, B. Zecca, P. Garred, M. G. De Simoni, the LEPAS group

**Affiliations:** Department of Neuroscience, IRCCS-Istituto di Ricerche Farmacologiche Mario Negri, Milan, Italy; Department of physiopathology and transplant, Milan University and Neuro ICU Fondazione IRCCS Cà Granda Ospedale Maggiore Policlinico, Milan, Italy; Emergency Care Unit, Fondazione IRCCS Cà Granda Ospedale Maggiore Policlinico, Milan, Italy; Angelo Bianchi Bonomi Haemophilia and Thrombosis Centre, Fondazione IRCCS Cà Granda Ospedale Maggiore Policlinico, Milan, Italy; Cerebrovascular Disease Unit, IRCCS-Istituto Neurologico Carlo Besta, Milan, Italy; Department of Neurology, San Gerardo Hospital, Milan Center for Neuroscience, University of Milano Bicocca, Monza, Italy; Department NESMOS, University of Rome “La Sapienza”, Rome, Italy; Valduce Hospital Como, Como, Italy; C. Mondino National Neurological Institute, Pavia, Italy; Division of Anesthesia and Intensive Care, Azienda Ospedaliero-Universitaria di Parma, Parma, Italy; Neurological Clinic, Marche Polytechnic University, Ancona, Italy; Laboratory of Moleclar Medicine, Department of Clinical Immunology, Section 7631, Rigshospitalet Faculty of Medical and Health Sciences, University of Copenhagen, Copenhagen, Denmark; Neurology Unit, Fondazione IRCCS Ca’ Granda Ospedale Maggiore Policlinico, Milan, Italy

**Keywords:** Ischemic stroke, Innate immunity, Ficolin-1, Ficolin-2, Ficolin-3, MBL, Lectin pathway

## Abstract

**Background:**

Several lines of evidence support the involvement of the lectin pathway of complement (LP) in the pathogenesis of acute ischemic stroke. The aim of this multicenter observational study was to assess the prognostic value of different circulating LP initiators in acute stroke.

**Methods:**

Plasma levels of the LP initiators ficolin-1, -2, and -3 and mannose-binding lectin (MBL) were measured in 80 stroke patients at 6 h only and in 85 patients at 48 h and later. Sixty-one age- and sex-matched healthy individuals served as controls. Stroke severity was measured on admission using the National Institutes of Health Stroke Scale (NIHSS). The outcome was measured at 90 days by the modified Rankin Scale (mRS).

**Results:**

Ficolin-1 was decreased in patients compared with controls measured at 6 h (median 0.13 vs 0.33 μg/ml, respectively, *p* < 0.0001). At 48 h, ficolin-1 was significantly higher (0.45 μg/ml, *p* < 0.0001) compared to the 6 h samples and to controls. Likewise, ficolin-2 was decreased at 6 h (2.70 vs 4.40 μg/ml, *p* < 0.0001) but not at 48 h. Ficolin-3 was decreased both at 6 and 48 h (17.3 and 18.23 vs 21.5 μg/ml, *p* < 0.001 and <0.05, respectively). For MBL no difference was detected between patients and controls or within patients at the different time points. In multivariate analysis, early ficolin-1 was independently associated with unfavorable mRS outcome (adjusted odds ratio (OR): 2.21, confidence interval (CI) 95 % 1.11–4.39, *p* = 0.023). Early ficolin-1 improved the discriminating ability of an outcome model including NIHSS and age (area under the curve (AUC) 0.95, CI 95 % 0.90–0.99, *p* = 0.0001).

**Conclusions:**

The ficolins are consumed within 6 h after stroke implicating activation of the LP. Early ficolin-1 is selectively related to 3-month unfavorable outcome.

**Electronic supplementary material:**

The online version of this article (doi:10.1186/s12974-016-0481-2) contains supplementary material, which is available to authorized users.

## Background

Blood biomarkers may play a relevant role in early stroke diagnosis, outcome prediction, or treatment, supporting clinicians in assessing severity and predict outcome. The quest for reliable stroke biomarkers, however, has been quite unsatisfactory, especially in the early phase. Among the inflammatory molecules contributing to ischemia, the ficolins, initiators of the lectin complement pathway (LP), may fulfill the criteria for reliable biomarkers [1, 2]. Ficolins (ficolin-1 or M-ficolin, ficolin-2 or L-ficolin and ficolin-3 or H-ficolin) are multimeric recognition molecules with high sequence similarities and binding specificity for structures exposed on the surface of pathogens and of injured host cells. They circulate in the blood associated with serine proteases (MASPs) and with non-enzymatic molecules named sMAP and MAP-1 [3]. Upon binding with their targets, ficolins activate the LP, promoting downstream complement activation. Ficolin-1 is primarily expressed in granulocytes and monocytes and is stored in secretory granules from where it is exocytosed to the bloodstream [4]. Once released, it binds back to the membrane of the granulocytes and monocytes in a calcium-dependent manner [5, 6]. As a consequence, the plasma levels of ficolin-1 are relatively low (0.3 μg/ml) [7]. Ficolin-2 and ficolin-3 are mainly produced by the liver and liver/lung and circulate at a median level of 5 and 25 μg/ml, respectively [8, 9]. In addition to the ficolins, three other pattern recognition molecules: mannose-binding lectin (MBL) and the recently identified collectins: collectin-10 (CL-L1 or CL-10) and collectin-11 (CL-K1 or CL-11) can also activate the LP [10–12]. The plasma concentration of these molecules varies among individuals, partly conditioned by specific gene molecular variants [13–15]. They also vary within individuals across time as a result of injury associated LP activation/consumption [1, 2, 4, 16]. Experimental and clinical evidence implicates a clear role of the LP in the progression of brain damage in stroke, and the data are consistent with an association of LP activation with unfavorable outcome [1, 14, 15, 17–19], with a few studies highlighting MBL [20, 21] and ficolin-3 [1] as independent predictors of outcome after ischemic stroke. The role of ficolin-1 and ficolin-2 has scarcely been studied [1, 2].

With the present study, we aimed at assessing the prognostic value of LP measurements and to analyze the involvement of LP after stroke in two cohorts of patients. In one of them, we analyzed the hyperacute changes in LP initiators. In the other, we assessed long-term changes.

## Methods

### Protocol approvals and patient consents

The study involved eight Italian stroke centers, with extensive experience in stroke diagnosis and treatment. The study was approved by the ethics committees of all participating centers. Written informed consent was obtained from each patient or his/her next of kin for the collection of data, blood samples, and subsequent analyses according to the study protocol and local rules (RS: 42/2011, Prot. C.E. 683/11).

### Study design and cohort description

**Fig. 1:**
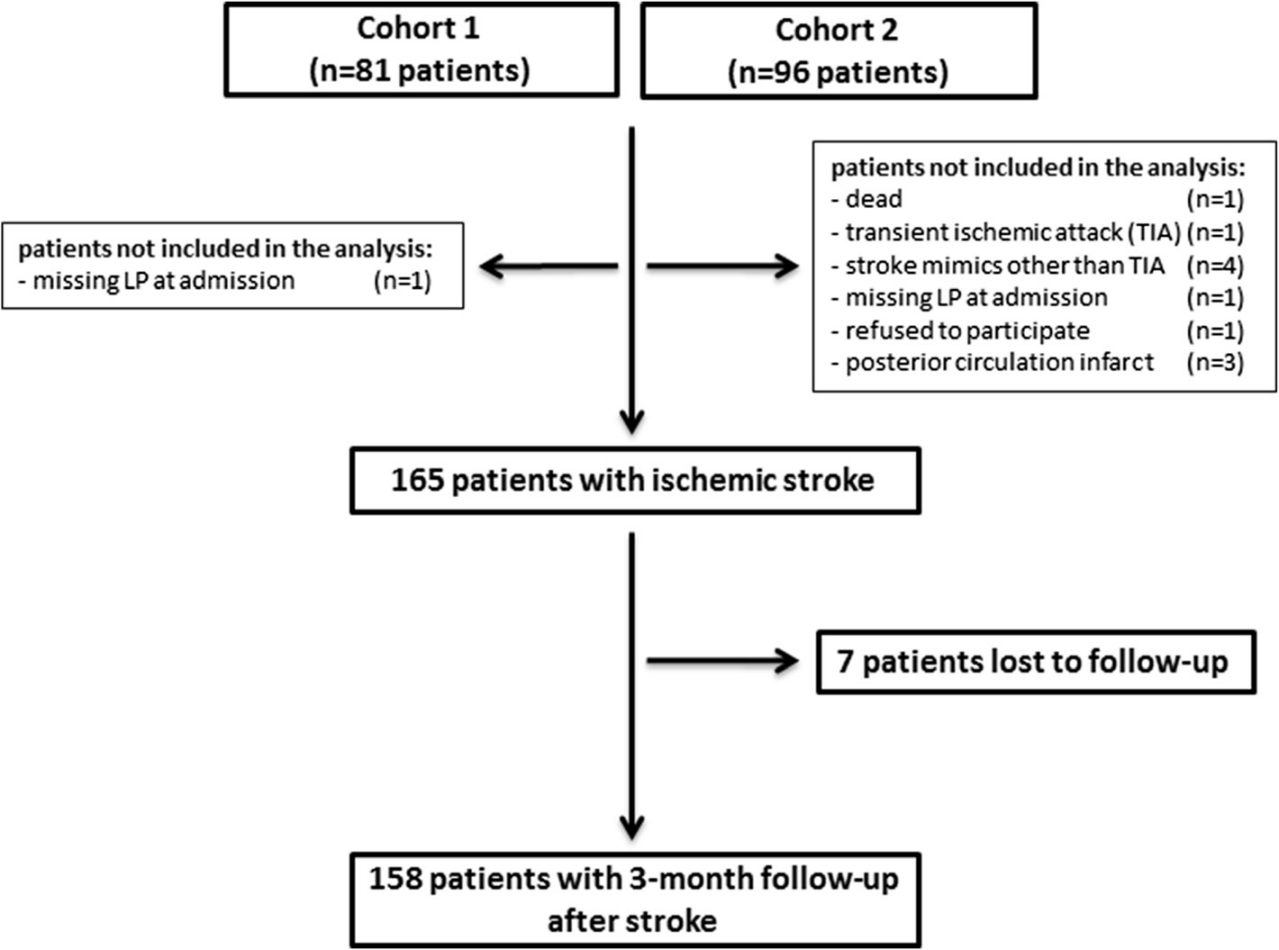
Patient flowchart

Two cohorts of patients were studied (flowchart, Fig. 1), the first was sampled within 6 h from symptom onset (80 patients, one recruiting center) and the second (85 patients, 8 recruiting center) was sampled longitudinally at 48 h, at 3–5 days, and at 1 month. Inclusion criteria were a clinical and radiologically confirmed diagnosis of first ischemic stroke event [22], age between 16 and 80 years, and absence of known complement deficiency (Fig. 1). Detailed demographic and clinical data, including stroke severity (National Institutes of Health Stroke Scale, NIHSS >14) [23] and etiology according to the TOAST (Trial of Org 10172 in Acute Stroke Treatment) classification [24], and conventional vascular risk factors and treatments were collected applying a standardized clinical form (Table 1).

**Table 1:** Baseline and clinical characteristics

	Controls (*n* = 61)	Patients	
		6 h (*n* = 80)	48 h (*n* = 85)
Demographic characteristics			
Age ≥ 50 years, *n* (%)	52 (85)	75 (94)	73 (86)
Gender, (M/F)	26/35	33/47	49/36
Race caucasian, *n* (%)	61 (100)	80 (100)	82 (96)
Risk factors, *n* (%)			
Hypertension	15 (24)	60 (75)***^, #^	60 (70)**
Diabetes	3 (5)	11 (14)	29 (34)***^, ##^
Dyslipidemia	12 (20)	43 (54)***	39 (46)
Cardiovascular diseases	2 (3)	24 (30)***	19 (22)
Atrial fibrillation	2 (3)	30 (37)***	15 (18)*
Smoking history	22 (37)	21 (26)	44 (57)
Recent Infections	2 (3)	–	1 (1)
CRP ≥ 3 mg/l	–	5 (6)	16 (19)
Clinical characteristics			
TOAST classification, *n* (%)			
Cardioembolism		24 (30)	23 (27)
Atherosclerosis		21 (26)	33 (39)
Small vessel occlusion		11 (14)	6 (7)
Undetermined etiology		24 (30)	22 (25)
Other determined etiology		–	2 (2)
NIHSS, median (IQR)		5 (2–11)	5 (2–11)
Severe (>14), *n* (%)		12 (16)	14 (17)
3-month mRS, median (IQR)		2 (1–4)	1 (0–3)
mRS (>2), *n* (%)		33 (41)	36 (42)
Mortality		10 (12)	2 (3)

Categorical variables are presented as number of patients with/without percentages in parentheses; continuous variables as median with interquartile range (IQR). Age was dichotomized using 50 years as cut-off (based on the minimum quartile in control group)

*TOAST* Trial of Org 10172 in Acute Stroke Treatment, *NIHSS* National Institutes of Health Stroke Scale, *3-month mRS* 3-month modified Rankin Scale, *CRP* C-reactive protein

*<0.05, **<0.01, ***<0.001, vs control group by univariate and ^#^<0.05, ^##^<0.01 by multivariate logistic regression analysis

Outcome evaluation, performed 3 months after stroke, included neurological examination and stroke disability assessment using the modified Rankin Scale (mRS, unfavorable outcome defined by mRS >2) [25]. Outcome was assessed by a neurologist blinded to the biochemical determinations. Controls consisted of 61 healthy individuals with no history of stroke. For each case, when possible, a control was selected by identifying a next of kin matched for age, race, and sex. Thirty-eight (23 %) patients received intravenous thrombolysis according to the European guidelines [26] and 127 (77 %) received a conservative treatment. In all patients from 6 h cohort, the blood sample was obtained in the hyperacute phase of stroke before rtPA treatment.

The coordinating center (Mario Negri Insitute) was responsible to supervise data collection. The research teams got in touch regularly (once every month) throughout the study to discuss progresses, including recruitment, withdrawals, and compliance. All the personnel involved fully understood the research protocol and standard operating procedures for the study.

### Blood sampling protocol

Protocols for sample collection and handling were identical for all centers. Clotting and complement activation were prevented by collecting samples in 10 mM of ethylenediaminetetraacetic acid (EDTA). Plasma was processed at 2000 *g* for 15 min at 4 °C and stored locally at each center at −80 °C before analysis. For myeloperoxidase (MPO) quantification, an additional centrifugation step was performed in order to avoid contamination with platelets and white blood cells. Analysis of plasma samples was centralized and performed at the University of Copenhagen and at the Mario Negri Institute. All plasma samples were thawed only once prior to use.

### Protein quantification

Ficolin-1, -2, and -3 and MBL assays were routinely determined by sandwich ELISAs using specific in-house produced monoclonal antibodies as previously described [7–9, 27]. All assays were optimized for automated analysis in the 384-well format on Biomek FX (Beckman Coulter, Fullerton, CA,USA) [25]. MPO was measured by a commercially available ELISA kit [28]. C-reactive protein (CRP) was determined by automated latex-enhanced immunoassay. Elevated baseline CRP (>3.0 mg/l) was used as marker of increased risk of sepsis [29].

### Other assays

D-dimer was assessed by automated latex-enhanced immunoassay [30]. Leukocyte count, percentage of neutrophils, percentage of lymphocytes, and neutrophils to lymphocytes ratio (N/L ratio) were determined on admission to the emergency department and within 24 h of the onset in 81.2 % of cases.

### Statistical analyses

Plasma concentration of complement components did not follow a normal distribution (*p* > 0.05, Shapiro-Wilk test). The two cohorts of patients were studied by indirect comparison analysis. Categorical variables were expressed as number of patients and proportions. Continuous variables were expressed as median and interquartile range (IQR) or mean and standard deviation (SD).

#### Univariate analysis

Baseline demographic and clinical characteristics between cases and controls and between patients were examined by means of the Fisher exact test for categorical variables and the Mann-Whitney *U* test for continuous variables. Age was analyzed as a continuous variable. The differences between groups and time points were compared using the Kruskal-Wallis test followed by Dunn post hoc test. Interactions between LP initiators and the potential confounders were examined by Wilcoxon-Mann-Whitney test and Spearman’s rank correlation coefficient (rho) for bivariate correlations between ficolin-1 and inflammatory markers.

#### Multivariate regression models and C-statistics

Multivariate analysis was performed by binary logistic regression analysis, including established risk factors and outcome predictors showing a significant univariate association. Significant predictors were tested for interaction, based on biological plausibility and on factors that might influence the prognostic value of LP initiators. The overall diagnostic accuracy of LP initiators was assessed with the area under the receiver operating characteristic (ROC) curve (AUC), with cut-offs obtained by pooling values for patients and controls. To examine whether the addition of LP markers improved the predictability of the clinical model for stroke outcome, a regression analysis by entering individual or a combined set of variables into the baseline clinical model (combined model assessed adjusting predicted values) was performed. The analysis was performed using age and NIHSS score as continuous variables. Odds ratio (OR) with 95 % confidence intervals (CI 95 %) was reported as measures of association. To account for data missing to follow-up, an additional analysis was performed assuming the “worst mRS scenario” for patients missing the 3-month evaluation. Statistical analysis was performed using Prism 5 (GraphPad software, San Diego, CA); SPSS 20.0 (SPSS Inc., Chicago, IL, USA), and SAS 9.2 (SAS Institute Inc., Cary, NC, USA).

## Results

### Baseline demographic and clinical characteristics

Patient enrollment and follow-up details are outlined in the flowchart (Fig. 1). The 3-month follow-up was recorded in 158 (96 %) patients. Mean age was 70 ± 13 (mean ± SD), and 50 % of patients were female. The median NIHSS was 9 (IQR 6–15), the median 3-month mRS and mortality were respectively 1 (IQR 0–4) and 11 (7 %). All demographic and clinical features of included patients from the two cohorts and controls are summarized in Table 1. As expected, at univariate analysis hypertension, diabetes, dyslipidemia, cardiovascular diseases, and atrial fibrillation were significantly more frequent in stroke patients than in controls (Table 1 and Additional file 1: Table S1). Missing values were as follows: smoking history, *n* = 2 (3 %) in controls and *n* = 8 (9 %) in patients enrolled within 48 h; NIHSS score, *n* = 3 (4 %); and mRS score, *n* = 7 (8 %), only in patients enrolled within 48 h. Possible confounding factors between the two cohorts were analyzed by multivariate analysis. The results showed similar sex predominance, stroke severity, stroke etiology, atrial fibrillation, functional outcome, and mortality and highlight differences for the prevalence of diabetes, smoking history, and elevated baseline C-reactive protein (Additional file 2: Table S2.1). Thus, we assessed possible changes in LP activators associated with these confounding factors (Additional file 2: Table S2.2). Notably, no interactions were found between ficolin-1 and the potential confounders. High ficolin-3 was associated with smoking history (nonsmokers vs smokers: 16.48 vs 20.15 ng/ml, *p* = 0.015), but multivariate analysis revealed no association between smoking and functional outcome (Table 2).

**Table 2:** Univariate and multivariate predictors of functional outcome

	6 h				48 h			
	Univariate		Multivariate		Univariate		Multivariate	
	OR (CI 95 %)	*p*	OR (CI 95 %)	*p*	OR (CI 95 %)	*p*	OR (CI 95 %)	*p*
Predictors								
Age	1.10 (1.03–1.17)	0.003	1.11 (1.04–1.19)	0.003	1.0 (0.96–1.04)	0.79	–	–
Gender	1.53 (0.60–3.90)	0.38	–	–	0.92 (0.36–2.39)	0.87	–	–
Hypertension	1.76 (0.59–5.27)	0.31	–	–	0.37 (0.13–1.02)	0.054	–	–
Diabetes	1.48 (0.39–5.61)	0.56	–	–	1.51 (0.57–3.98)	0.40	–	–
Dyslipidemia	0.80 (0.32–1.98)	0.63	–	–	0.68 (0.26–1.75)	0.42	–	–
Cardiovascular diseases	0.60 (0.22–1.65)	0.33	–	–	1.23 (0.27–5.58)	0.79	–	–
Atrial fibrillation	2.17 (0.84–5.60)	0.11	–	–	1.0 (0.30–3.30)	1.0	–	–
Smoking history	0.44 (0.15–1.25)	0.19	–	–	1.08 (0.38–3.07)	1.0	–	–
Toast classification	0.68 (0.46–1.01)	0.055	–	–	1.08 (0.81–1.43)	0.59	–	–
NIHSS	1.17 (1.06–1.29)	0.002	1.17 (1.05–1.31)	0.005	1.14 (1.05–1.24)	0.002	1.14 (1.05–1.24)	0.002
Ficolin-1^a^	1.73 (1.03–2.91)	0.039	2.21 (1.11–4.39)	0.023	0.52 (0.03–9.08)	0.65	–	–
Ficolin-3	0.99 (0.93–1.05)	0.76	–	–	0.93 (0.85–1.01)	0.11	–	–

Exact *p* value for univariate and multivariate logistic regression analysis is reported

*CI 95 %* 95 % confidence interval, *OR* odds ratio, *NIHSS* National Institutes of Health Stroke Scale, *3-month mRS* 3-month modified Rankin Scale

^a^Note that the odds ratio corresponds to a 0.1 unit change in the explanatory variable

### LP initiators in healthy controls and acute ischemic stroke patients

**Fig. 2:**
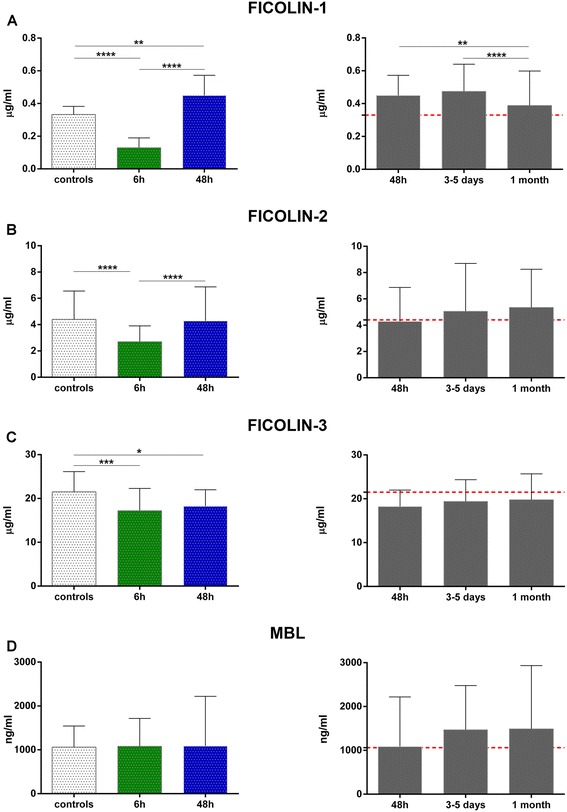
LP initiators at early and late time points. Plasma concentrations of ficolin-1 (**a**), ficolin-2 (**b**), ficolin-3 (**c**), and MBL (**d**) in controls (*n* = 61) and in two different group of stroke patients sampled at different time points after stroke (*n* = 80 patients within 6 h and *n* = 85 within 48 h). In the latter group, blood samples were collected at baseline, at days 3–5 (*n* = 78) and at 1 month (*n* = 60) after stroke. The *dotted red line* indicates the median value in controls. Data are expressed as median with interquartile range. *p* values *<0.05, **<0.01, ***<0.001, ****<0.0001 versus control and among groups, Kruskal-Wallis test with Dunn post hoc test

Plasma levels of ficolin-1 were significantly lower in patients compared to controls when measured within 6 h (median 0.13 vs 0.33 μg/ml, respectively, *p* < 0.0001; Fig. 2a). At 48 h, ficolin-1 levels were significantly higher (0.45 μg/ml; Fig. 2a) compared to the 6 h cohort and to controls. Ficolin-2 levels were decreased at 6 h, but not at 48 h after stroke, in patients versus controls (2.70 vs 4.40 μg/ml, respectively, *p* < 0.0001; Fig. 2b). Ficolin-3 levels were significantly lower both at 6 and 48 h (patients: 17.30 and 18.23 vs controls: 21.50 μg/ml, *p* < 0.001 and <0.05, respectively; Fig. 2c). Twenty-two patients (13 %) and five controls (8 %) had MBL levels <100 ng/ml, a value associated with MBL deficiency [31]. Grouping patients according to this cut-off resulted in no differences in demographics and clinical characteristics (data not shown). No significant differences were detected for MBL levels between patients at 6 and 48 h and controls (patients: 1081 and 1085 vs controls: 1060 ng/ml, *p* > 0.05, respectively; Fig. 2d).

The time-course analysis showed that the increased ficolin-1 concentrations persisted at least up to 3–5 days after stroke (controls: 0.33 vs patients: 0.47 μg/ml, *p* < 0.01; Fig. 2a) with no changes in the other LP initiators. Admission levels of ficolins and MBL were not related to initial stroke severity, defined according to the NIHSS score, or to different stroke etiology (data not shown).

### Diagnostic accuracies of LP initiators for discriminating stroke patients from controls

**Fig. 3:**
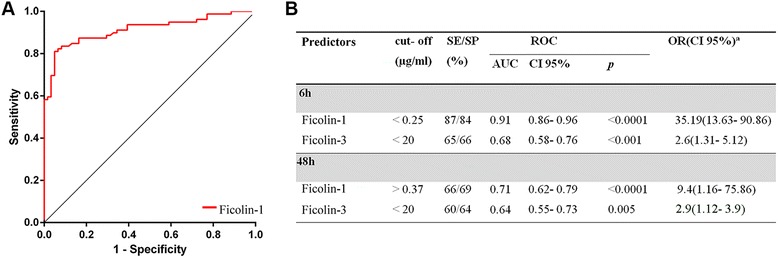
Diagnostic accuracy of ficolin-1 and ficolin-3 in discriminating stroke patients from controls. ROC curve of early ficolin-1 levels (6 h) demonstrating sensitivity as a function of 1-specificity for discriminating case/control status at the early time point (**a**). ROC analysis data, including optimal cut-offs, of ficolin-1 and ficolin-3 for discriminating case/control status at early and late time points (**b**). The AUC and exact *p* value for asymptotic significance are reported. *SE* sensitivity, *SP* specificity, *AUC* area under the curve, *CI 95 %* 95 % confidence interval, *OR* odds ratio. ^a^The odds ratio corresponds to a unit change in the explanatory categorical variables

We found that the AUCs of ficolin-1 and ficolin-3 had good diagnostic accuracy (ficolin-1: 0.91, *p* < 0.0001; ficolin-3: 0.68, *p* < 0.001; Fig. 3a, b). However, early ficolin-1 was significantly better in discriminating the ischemic condition compared to delayed ficolin-1 or ficolin-3 values (Fig. 3b). The optimal cut-off value of ficolin-1 as a diagnostic marker of stroke was projected to be 0.25 μg/ml, which yielded a sensitivity of 87 % and a specificity of 84 %; at this cut-off, the odds ratio (OR) was 35.19 [95 % confidence intervals (CI 95 %) 13.63–90.86, *p* < 0.0001].

### Prediction of functional outcome at 3 months

**Fig. 4:**
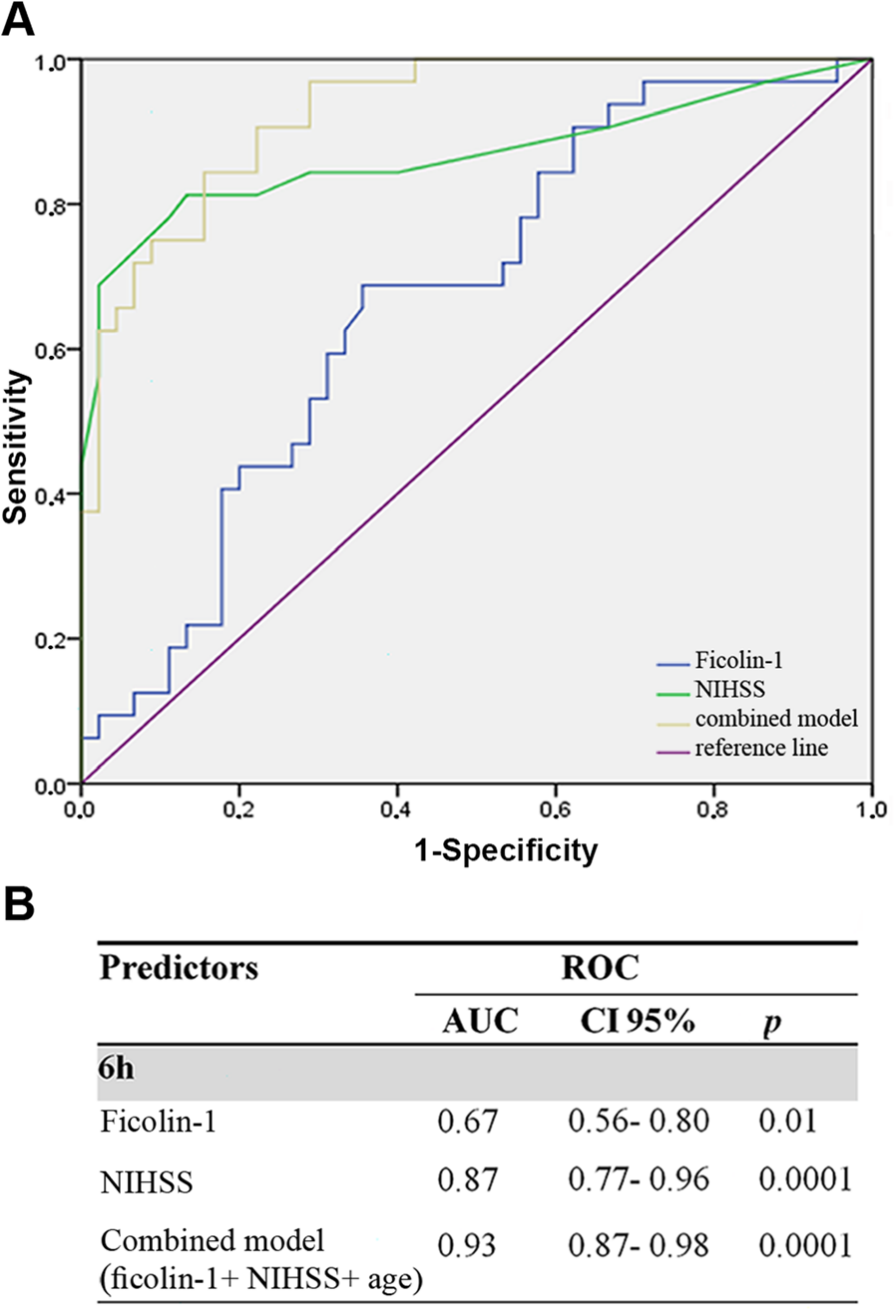
Prognostic accuracies of early ficolin-1 as predictor of unfavorable outcome. ROC curve of early ficolin-1 levels (6 h) demonstrating sensitivity as a function of 1-specificity for predicting functional outcome at 3 months, based on the logistic model incorporating the relative contribution of each predictor in the combined model (ficolin-1 adjusted predictive values for NIHSS and age (**a**). The AUC and exact *p* value for asymptotic significance are reported (**b**). *AUC* area under the curve, *CI 95 %* 95 % confidence interval, *NIHSS* National Institutes of Health Stroke Scale

A total of 69 patients (43 %) had an unfavorable outcome at 3 months. In the univariate model, 6 h ficolin-1 (OR 1.73, CI 95 % 1.03–2.91, *p* = 0.039), age (OR 1.10, CI 95 % 1.03–1.17, *p* = 0.003), and NIHSS score (OR 1.17, CI 95 %: 1.06–1.29, *p* = 0.002) were associated with unfavorable outcome. Subsequent multivariate analysis demonstrated that 6 h ficolin-1 was independently predictive of an unfavorable outcome after adjustment for all other significant outcome predictors (adjusted OR 2.21, CI 95 % 1.11–4.39, *p* = 0.023; Table 2). The addition of 6 h ficolin-1 to a combined clinical model including NIHSS score and age further improved the discriminatory accuracy of the NIHSS [AUC 0.93(CI 95 % 0.87–0.98), *p* = 0.0001; AUC of the NIHSS 0.87 (CI 95 % 0.77–0.96); Fig. 4a, b]. Notably, 6 h ficolin-1 had a similar crude prognostic accuracy as compared with the reference markers of functional outcome CRP and d-dimer [32] (Additional file 3: Table S3). When measured at 48 h, ficolin-1 was no longer associated with unfavorable outcome whereas NIHSS score was found to be predictive of long-term functional outcome (Table 2). Seven patients were lost to follow-up; assuming that these patients had the worst scenario, no changes were found in ficolin levels (Additional file 4: Table S4).

**Fig. 5:**
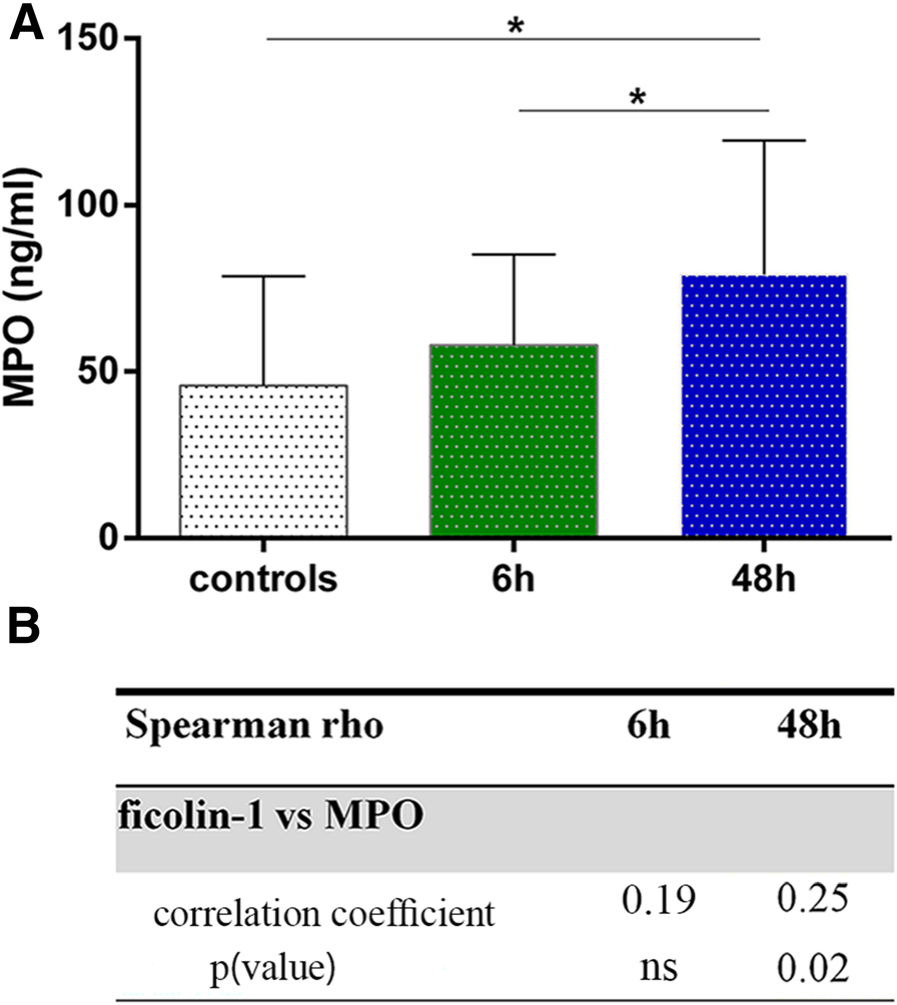
MPO levels at early and late time points in stroke patients and controls. Plasma concentrations of MPO in controls and patients with stroke (sampled within 6 h and 48 h, respectively). Data are expressed as median with interquartile range. *p* values *<0.05 versus control and among groups, Kruskal-Wallis test with Dunn post hoc test (**a**). Spearman’s rho for ficolin-1 versus MPO (**b**). *MPO* myeloperoxidase

Because peripheral blood cells of myeloid lineage are the first source of circulating ficolin-1 [6, 33–35], we measured MPO as marker of secretory vesicle mobilization [6]. In stroke patients, MPO levels at 6 h where not different compared to controls (57.99 vs 45.95 ng/ml, respectively, *p* > 0.05; Fig. 5a). Notably, MPO levels were higher in 48 h than in 6 h patients (79.20 vs 57.99 ng/ml, *p* < 0.05; Fig. 5a). Ficolin-1 MPO correlation was not present at 6 h, but a slight correlation was observed at 48 h (*r* = 0.25, *p* = 0.02; Fig. 5b). In accordance with this, 48 h ficolin-1 values were significantly correlated with total leukocyte count (*p* = 0.008), neutrophil count, and N/L ratio (*p* = 0.08), whereas lymphocytes were negatively correlated (*p* = 0.003; Additional file 5: Table S5). A total of 12 patients (7 %) died within 3 months after stroke. Due to the low number of deaths, the overall predictive value ability of ficolin-1 to distinguish survivors from non-survivors could not be estimated.

## Discussion

The LP, now recognized to be at the crossroad between complement and coagulation, is known to be deeply involved in the pathophysiology of brain ischemic injury [1, 2, 14, 15, 17, 20, 21, 36]. The present multicenter observational study shows that LP in plasma is already consumed 6 h following stroke, consistent with LP activation, and that among the LP initiators evaluated in this study, ficolin-1 is selectively related to an unfavorable outcome 3 months after ischemic stroke. Our data demonstrate for the first time that ficolin-1 is an independent predictor of functional outcome capable of improving the discriminatory ability of the NIHSS and of age in a combined prognostic model. Ficolin-1 is a recently identified LP activator. It is synthesized and presented on the surface of peripheral monocytes and neutrophils, possibly promoting neutrophil adhesion, aggregation, and migration [4, 6, 33–35, 37]. Because of these characteristics, ficolin-1 might play a role in ischemic damage; however, no data were available up to now in stroke patients. As a possible consequence of its presence on cells, its circulating levels are relatively low [7, 38]. Consistently with previous data [7], our results show that in healthy subjects, ficolin-1 circulates in plasma and is present at a median concentration of 0.33 μg/ml. Compared to controls, ficolin-1 levels are significantly lower when measured within 6 h after stroke and significantly higher when measured at longer time points, up to 3–5 days. It can be hypothesized that at the early time point, when plasma ficolin-1 levels are associated to 3-month outcome, consumption exceeds production/release and plasma levels essentially reflect the degree of local brain inflammatory response [33]. Later on, when ficolin-1 production/release exceeds consumption, the plasma concentration increases. The lack of association between ficolin-1 and the NIHSS, an index of neurological impairment, suggests that, besides the degree of initial injury, an overwhelming acute inflammatory host response significantly contributes to the pathogenesis of stroke and long-term outcome. Interestingly, at late sampling time point (from 48 h on), but not earlier, ficolin-1 correlates with MPO suggesting that at this later stage, both parameters reflect the overall leukocyte activation. This is also mirrored by the correlation with the neutrophil count at the same late sampling time. Not surprisingly, at this time point, ficolin-1 has lost its prognostic value. This indicates that ficolin-1 behavior is not the typical one of an acute phase protein. A similar behavior in a different contest has been reported in patients undergoing colorectal cancer surgery who display an early reduction of ficolin-1, followed by a rebound at longer time points (up to 2 weeks) with no relation to CRP changes [38].

As compared to healthy subjects, ficolin-2 plasma levels were significantly lower in stroke patient samples obtained within 6 h after the onset of symptoms. This finding is similar to that reported by Füst and collaborators 8 h after stroke [1]. They showed decreased ficolin-2 levels that persisted up to 3 days even though with a high variability. In our study, 3–5 days samples revealed a complete recovery to normal values. Interestingly, an early decrease in ficolin-2 was also observed in subarachnoid hemorrhage patients [2]. In the present study, ficolin-2 levels were not associated with stroke severity indicating that this protein may be related to a systemic inflammatory response rather than to local brain events.

We observed also an early and persistent consumption of ficolin-3. This is in agreement with previous observations [1]. This LP activator shows a significant diagnostic accuracy in discriminating patients from controls, although to a lower extent than ficolin-1. The prolonged reduction suggests that in these patients, ficolin-3 is used over time. Ficolin-3 was previously reported to be associated with an unfavorable prognosis when assessed at days 3–4, but not at 8 h [1]. A recent study shows that ficolin-3 functional LP activity, but not its levels, was associated with unfavorable outcome in subarachnoid hemorrhage patients [2]. The discrepancies between the different studies are not clear and could indicate heterogeneity or subtle clinical differences between the different cohorts. Nevertheless, taken together, these data implicate a role for ficolin-3 in stroke although its specific prognostic value still needs to be clarified.

In line with the reported prevalence [13], approximately 13 % of our patients show low MBL concentrations (<100 ng/ml). The clinical characteristics of patients with MBL <100 ng/ml and those with levels >100 ng/ml did not differ, and we did not find evidence of MBL changes related to stroke severity or outcome. Genetically defined MBL deficiency has been associated with a better outcome after stroke [14, 15]. Consistently, MBL production has been correlated with increased risk of acute stroke [36], morbidity, and mortality after stroke [20, 21]. The circulating levels measured here, being the result of a balance between genetic and pathophysiological conditions, may conceal the actual relationship between MBL and stroke. The lack of correlation could indicate that previous observed associations with MBL are related to heterogeneity among different stroke cohorts. Thus, further studies, including the detection of genotype of the patients are necessary to elucidate the role of MBL in the pathogenesis of stroke. Nevertheless, the available studies indicate that the different LP molecules emerge as sensitive biomarkers, which may reflect underlying pathophysiology and outcome of subarachnoid hemorrhage and ischemic stroke.

This study has some limitations. First, the plasma samples analyzed were obtained from two cohorts with different sampling time (within 6 h in the first group and within 48 h in the other one). The investigation of different patients at different times was simply due to the participation of the Emergency Room staff (the one charged with 6 h sampling), which could be confirmed only in one center. Thus, the indirect comparison allows only to hypothesize the temporal changes of ficolin levels within 48 h after stroke onset. A confirmatory longitudinal study with a larger sample size will be therefore necessary to exactly define the window of interest of ficolin-1 changes. The short-term changes over time were not available to test intra-individual time trends. In addition, it should be mentioned that we did not analyze the significance of LP initiators in patients undergoing thrombolysis since our study was underpowered to meet this aim. A dedicated study should be performed to understand if and how thrombolytics may affect lectin protein consumption.

## Conclusions

In conclusion, our study shows that the ficolins are consumed within 6 h after ischemic stroke and identifies for the first time ficolin-1 as a sensitive prognostic marker for stroke. Ischemic stroke is a heterogeneous disorder, and efforts are needed to better define its molecular biology. The available data indicate that LP is involved to a different extent and with distinctive functions over time in ischemic and hemorrhagic stroke. We now provide evidence of a specific role for ficolin-1 showing higher sensitivity compared to the other LP activators towards stroke outcome.

## Additional files

Additional file 1: Table S1. Multivariate analyses for stroke diagnosis. (PDF 77 kb) Additional file 2: Table S2.1. Baseline and clinical characteristics between cohorts. **Table S2.2.** Ficolins and MBL vs confounding factors. (PDF 170 kb) Additional file 3: Table S3. Comparison of the crude performance of early ficolin-1 and diagnostic markers of functional outcome in patients enrolled within 6 h. (PDF 12 kb) Additional file 4: Table S4. Univariate and multivariate predictors of functional outcome assuming the worst scenario for patients lost to follow-up. (PDF 88 kb) Additional file 5: Table S5. Leukocyte count in 48 h cohort. (PDF 64 kb)

## Abbreviations

CRP: C-reactive protein
LP: lectin pathway of complement
MBL: mannose-binding lectin
MPO: myeloperoxidase
mRS: modified Rankin Scale
N/L ratio: neutrophil to lymphocyte ratio
NIHSS: National Institutes of Health Stroke Scale
rtPA: recombinant tissue plasminogen activator
TOAST: Trial of Org 10172 in Acute Stroke Treatment

## References

[1] Füst G, Munthe-Fog L, Illes Z, Széplaki G, Molnar T, Pusch G (2011). Low ficolin-3 levels in early follow-up serum samples are associated with the severity and unfavorable outcome of acute ischemic stroke. J Neuroinflammation.

[2] Zanier ER, Zangari R, Munthe-Fog L, Hein E, Zoerle T, Conte V (2014). Ficolin-3-mediated lectin complement pathway activation in patients with subarachnoid hemorrhage. Neurology.

[3] Yongqing T, Drentin N, Duncan RC, Wijeyewickrema LC, Pike RN (2012). Mannose-binding lectin serine proteases and associated proteins of the lectin pathway of complement: two genes, five proteins and many functions?. Biochim Biophys Acta.

[4] Endo Y, Matsushita M, Fujita T (2015). New insights into the role of ficolins in the lectin pathway of innate immunity. Int Rev Cell Mol Biol.

[5] Honoré C, Rørvig S, Hummelshøj T, Skjoedt M-O, Borregaard N, Garred P (2010). Tethering of Ficolin-1 to cell surfaces through recognition of sialic acid by the fibrinogen-like domain. J Leukoc Biol.

[6] Rørvig S, Honore C, Larsson L-I, Ohlsson S, Pedersen CC, Jacobsen LC (2009). Ficolin-1 is present in a highly mobilizable subset of human neutrophil granules and associates with the cell surface after stimulation with fMLP. J Leukoc Biol.

[7] Munthe-Fog L, Hummelshoj T, Honoré C, Moller ME, Skjoedt MO, Palsgaard I (2012). Variation in FCN1 affects biosynthesis of ficolin-1 and is associated with outcome of systemic inflammation. Genes Immun.

[8] Munthe-Fog L, Hummelshøj T, Hansen BE, Koch C, Madsen HO, Skjødt K (2007). The impact of FCN2 polymorphisms and haplotypes on the Ficolin-2 serum levels. Scand J Immunol.

[9] Munthe-Fog L, Hummelshøj T, Ma YJ, Hansen BE, Koch C, Madsen HO (2008). Characterization of a polymorphism in the coding sequence of FCN3 resulting in a Ficolin-3 (Hakata antigen) deficiency state. Mol Immunol.

[10] Kawasaki N, Yokota Y, Kawasaki T (1993). Differentiation of conglutination activity and sugar-binding activity of conglutinin after removal of NH2-terminal 54 amino acid residues by endogenous serine protease(s). Arch Biochem Biophys.

[11] Axelgaard E, Jensen L, Dyrlund TF, Nielsen HJ, Enghild JJ, Thiel S (2013). Investigations on collectin liver 1. J Biol Chem.

[12] Ma YJ, Skjoedt M-O, Garred P (2013). Collectin-11/MASP complex formation triggers activation of the lectin complement pathway–the fifth lectin pathway initiation complex. J Innate Immun.

[13] Garred P, Larsen F, Seyfarth J, Fujita R, Madsen HO (2006). Mannose-binding lectin and its genetic variants. Genes Immun.

[14] Cervera A, Planas AM, Justicia C, Urra X, Jensenius JC, Torres F (2010). Genetically-defined deficiency of mannose-binding lectin is associated with protection after experimental stroke in mice and outcome in human stroke. PloS One.

[15] Osthoff M, Katan M, Fluri F, Schuetz P, Bingisser R, Kappos L (2011). Mannose-binding lectin deficiency is associated with smaller infarction size and favorable outcome in ischemic stroke patients. PloS One.

[16] Orsini F, De Blasio D, Zangari R, Zanier ER, De Simoni M-G (2014). Versatility of the complement system in neuroinflammation, neurodegeneration and brain homeostasis. Front Cell Neurosci.

[17] Orsini F, Villa P, Parrella S, Zangari R, Zanier ER, Gesuete R (2012). Targeting mannose-binding lectin confers long-lasting protection with a surprisingly wide therapeutic window in cerebral ischemia. Circulation.

[18] De la Rosa X, Cervera A, Kristoffersen AK, Valdés CP, Varma HM, Justicia C (2014). Mannose-binding lectin promotes local microvascular thrombosis after transient brain ischemia in mice. Stroke J Cereb Circ.

[19] Széplaki G, Szegedi R, Hirschberg K, Gombos T, Varga L, Karádi I (2009). Strong complement activation after acute ischemic stroke is associated with unfavorable outcomes. Atherosclerosis.

[20] Song F-Y, Wu M-H, Zhu L-H, Zhang Z-Q, Qi Q-D, Lou C-L (2015). Elevated serum mannose-binding lectin levels are associated with poor outcome after acute ischemic stroke in patients with type 2 diabetes. Mol Neurobiol.

[21] Zhang Z-G, Wang C, Wang J, Zhang Z, Yang Y-L, Gao L (2015). Prognostic value of mannose-binding lectin: 90-day outcome in patients with acute ischemic stroke. Mol Neurobiol.

[22] WHO MONICA Project Principal Investigators (1988). The World Health Organization MONICA Project (monitoring trends and determinants in cardiovascular disease): a major international collaboration. WHO MONICA Project Principal Investigators. J Clin Epidemiol.

[23] Brott T, Adams HP, Olinger CP, Marler JR, Barsan WG, Biller J (1989). Measurements of acute cerebral infarction: a clinical examination scale. Stroke J Cereb Circ.

[24] Adams HP, Bendixen BH, Kappelle LJ, Biller J, Love BB, Gordon DL (1993). Classification of subtype of acute ischemic stroke. Definitions for use in a multicenter clinical trial. TOAST. Trial of Org 10172 in Acute Stroke Treatment. Stroke J Cereb Circ.

[25] Hein E, Munthe-Fog L, Thiara AS, Fiane AE, Mollnes TE, Garred P (2015). Heparin-coated cardiopulmonary bypass circuits selectively deplete the pattern recognition molecule ficolin-2 of the lectin complement pathway in vivo. Clin Exp Immunol.

[26] European Stroke Organisation (ESO) Executive Committee, ESO Writing Committee (2008). Guidelines for management of ischaemic stroke and transient ischaemic attack 2008. Cerebrovasc Dis Basel Switz.

[27] Garred P, Madsen HO, Kurtzhals JA, Lamm LU, Thiel S, Hey AS (1992). Diallelic polymorphism may explain variations of the blood concentration of mannan-binding protein in Eskimos, but not in black Africans. Eur J Immunogenetics Off J Br Soc Histocompat Immunogenetics.

[28] Zanier ER, Brandi G, Peri G, Longhi L, Zoerle T, Tettamanti M (2011). Cerebrospinal fluid pentraxin 3 early after subarachnoid hemorrhage is associated with vasospasm. Intensive Care Med.

[29] Wang HE, Shapiro NI, Safford MM, Griffin R, Judd S, Rodgers JB (2013). High-sensitivity C-reactive protein and risk of sepsis. PloS One.

[30] Zecca B, Mandelli C, Maino A, Casiraghi C, Bolla G, Consonni D (2014). A bioclinical pattern for the early diagnosis of cardioembolic stroke. Emerg Med Int.

[31] Eisen DP, Dean MM, Boermeester MA, Fidler KJ, Gordon AC, Kronborg G (2008). Low serum mannose-binding lectin level increases the risk of death due to pneumococcal infection. Clin Infect Dis Off Publ Infect Dis Soc Am.

[32] Whiteley W, Tian Y, Jickling GC (2012). Blood biomarkers in stroke: research and clinical practice. Int J Stroke Off J Int Stroke Soc.

[33] Liu Y, Endo Y, Iwaki D, Nakata M, Matsushita M, Wada I, Inoue K, Munakata M, Fujita T: Human M-ficolin is a secretory protein that activates the lectin complement pathway. J Immunol Baltim Md 1950. 2005;175:3150–6. 10.4049/jimmunol.175.5.3150 16116205

[34] Honoré C, Rørvig S, Munthe-Fog L, Hummelshøj T, Madsen HO, Borregaard N (2008). The innate pattern recognition molecule Ficolin-1 is secreted by monocytes/macrophages and is circulating in human plasma. Mol Immunol.

[35] Zhang J, Yang L, Ang Z, Yoong SL, Tran TTT, Anand GS, Tan NS, Ho B, Ding JL: Secreted M-ficolin anchors onto monocyte transmembrane G protein-coupled receptor 43 and cross talks with plasma C-reactive protein to mediate immune signaling and regulate host defense. J Immunol Baltim Md 1950. 2010;185:6899–910. 10.4049/jimmunol.1001225 21037097

[36] Wang Z-Y, Sun Z-R, Zhang L-M (2014). The relationship between serum mannose-binding lectin levels and acute ischemic stroke risk. Neurochem Res.

[37] Hummelshoj T, Fog LM, Madsen HO, Sim RB, Garred P (2008). Comparative study of the human ficolins reveals unique features of Ficolin-3 (Hakata antigen). Mol Immunol.

[38] Wittenborn T, Thiel S, Jensen L, Nielsen HJ, Jensenius JC (2010). Characteristics and biological variations of M-ficolin, a pattern recognition molecule, in plasma. J Innate Immun.

