# Supplementary material for: Early ficolin-1 is a sensitive prognostic marker for functional outcome in ischemic stroke
Source: J Neuroinflammation. 2016 Jan 20;13:16. doi: 10.1186/s12974-016-0481-2 (PMC4721111; doi:10.1186/s12974-016-0481-2)
Supplement: Additional file 1: Table S1. — Multivariate analyses for stroke diagnosis. (PDF 77 kb) [file 12974_2016_481_MOESM1_ESM.pdf]

Variables associated with the diagnosis of stroke were first assessed by univariate analysis. Using multivariate analysis the combined predictors were tested to identify independent predictors of stroke, as detailed in table S1. Hypertension and diabetes were independently associated with stroke event. Low ficolin-3 was associated with hypertension (no- hypertension vs hypertension: 19.28 vs 16.82 ng/ml, *p*= 0.05) in 6h cohort. After adjusting for significant predictors, ficolin-3 remained as an independent stroke predictor (adjusted AUC: 0.68, CI 95%: 0.58–0.76) as shown in crude AUC (Fig. 3). No interaction was found between LP initiator levels and diabetes (data not shown).

Table S1. Multivariate analyses for stroke diagnosis

	**6h**		**48h**	
	**OR(CI 95%)[a]**	***p***	**OR(CI 95%)[a]**	***p***
**Predictors**				
Hypertension	**3.63(1.25- 10.53)**	**0.02**	2.24(0.77- 6.44)	0.13
Diabetes	2.92(0.31- 27.24)	0.35	**20.54(2.36- 179.13)**	**0.006**
Dyslipidemia	1.11(0.40- 3.08)	0.83	1.93(0.72- 5.22)	0.19
[b]Cardiovascular diseases	-	0.99	-	0.99
Atrial fibrillation	4.13(0.99- 17.17)	0.51	3.32(0.60- 18.34)	0.17

Multivariate analysis of significant univariate predictors of stroke in patients with stroke (sampled within 6h and 48h, respectively). Exact *p* value for multivariate logistic regression analysis is reported. 95% Confidence Interval (CI 95%); Odds Ratio (OR). [a]Note that the odds ratio corresponds to a unit change in the explanatory variable. [b]The odds ratio corresponds to 1.
