# Supplementary material for: Early ficolin-1 is a sensitive prognostic marker for functional outcome in ischemic stroke
Source: J Neuroinflammation. 2016 Jan 20;13:16. doi: 10.1186/s12974-016-0481-2 (PMC4721111; doi:10.1186/s12974-016-0481-2)
Supplement: Additional file 2: Table S2.1. — Baseline and clinical characteristics between cohorts. Table S2.2. Ficolins and MBL vs confounding factors. (PDF 170 kb) [file 12974_2016_481_MOESM2_ESM.pdf]

Table S2.1. Baseline and clinical characteristics between cohorts

	6h (n=80)	48h (n=85)	univariate	multivariate
			p (value)	*p* (value)
**Demographic characteristics**				
Age ≥ 50 y, *n (%)*	75(94)	73(86)	0.126	-
Gender, (M/F)	33/47	49/36	**0.043**	0.768
Race caucasian, *n (%)*	80(100)	82(96)	0.246	-
**Risk factors, *n (%)***				
Hypertension	60(75)	60(70)	0.601	-
Diabetes	11(14)	29(34)	**0.003**	**0.003**
Dyslipidemia	43(54)	39(46)	0.352	-
Cardiovascular Diseases	24(30)	19(22)	0.291	-
Atrial Fibrillation	30(37)	15(18)	**0.007**	0.118
Smoking History	21(26)	44(57)	**0.001**	**0.008**
Recent Infections	-	1(1)	-	**-**
CRP ≥ 3 mg/l	5(6)	16(19)	**0.019**	**0.018**
**Clinical characteristics**				
**TOAST Classification, n(%)**				
Cardioembolism	24(30)	23(27)	0.731	-
Atherosclerosis	21(26)	33(39)	0.098	-
Small Vessel Occlusion	11(14)	6(7)	0.296	-
Undetermined Etiology	24(30)	22(25)	0.604	-
Other Determined Etiology	-	2(2)	-	-
**NIHSS, Median (IQR)**	5(2- 11)	5(2- 11)		
Severe (>14), *n(%)*	12(16)	14(17)	1	-
**3-month mRS, median (IQR)**	2(1- 4)	1(0- 3)		
mRS (>2), n(%)	33(41)	36(42)	0.274	-
Mortality	10(12)	2(3)	**0.036**	0.999

TOAST: Trial of Org 10172 in Acute Stroke Treatment; NIHSS: National Institutes of Health Stroke Scale; 3- month mRS: 3- month modified Rankin scale. CRP: C-reactive protein. Categorical variables are presented as number of patients with/without percentages in parentheses; continuous variables as median with interquartile range (IQR). Age is presented as dichotomized using 50 years as cut-off (based on the minimum quartile in control group). Exact *p* value between cohorts by univariate and multivariate logistic regression analysis.

Table S2.2. Ficolins and MBL vs confounding factors

	**Ficolin-1**	**Ficolin-2**	**Ficolin-3**	**MBL**
6h				
Diabetes	0.265	0.322	0.700	0.831
Smoking History	0.536	0.509	0.919	0.461
CRP ≥ 3 mg/l	0.1	0.144	0.437	0.125
48h				
Diabetes	0.225	0.364	0.330	0.983
Smoking History	0.140	0.120	**0.015**	0.747
CRP ≥ 3 mg/l	0.169	0.677	0.791	0.733

Exact *p* value by Wilcoxon-Mann-Whitney test.
