# Supplementary material for: Early ficolin-1 is a sensitive prognostic marker for functional outcome in ischemic stroke
Source: J Neuroinflammation. 2016 Jan 20;13:16. doi: 10.1186/s12974-016-0481-2 (PMC4721111; doi:10.1186/s12974-016-0481-2)
Supplement: Additional file 3: Table S3. — Comparison of the crude performance of early ficolin-1 and diagnostic markers of functional outcome in patients enrolled within 6 h. (PDF 12 kb) [file 12974_2016_481_MOESM3_ESM.pdf]

Table S3. Comparison of the crude performance of early ficolin-1 and diagnostic markers of functional outcome in patients enrolled within 6h

**Predictors**	**ROC**		
	**AUC**	**CI 95%**	***p***
**6h**			
CRP	0.72	0.60- 0.83	0.001
D-dimer	0.65	0.52- 0.79	0.02
Ficolin-1+ CRP	0.69	0.57- 0.81	0.012
Ficolin-1 + D-Dimer	0.66	0.53- 0.79	0.005
Ficolin-1+ CRP+D-dimer	0.73	0.60- 0.84	0.001

ROC analysis data of ficolin-1, C-reactive protein (CRP) and D-dimer. The AUC and exact *p* value for asymptotic significance are reported. Area Under the Curve (AUC); 95% Confidence Interval (CI 95%).
