# Supplementary material for: Early ficolin-1 is a sensitive prognostic marker for functional outcome in ischemic stroke
Source: J Neuroinflammation. 2016 Jan 20;13:16. doi: 10.1186/s12974-016-0481-2 (PMC4721111; doi:10.1186/s12974-016-0481-2)
Supplement: Additional file 4: Table S4. — Univariate and multivariate predictors of functional outcome assuming the worst scenario for patients lost to follow-up. (PDF 88 kb) [file 12974_2016_481_MOESM4_ESM.pdf]

Table S4. Univariate and multivariate predictors of functional outcome assuming the worst scenario for patients lost to follow up

	**48h**			
	**univariate**		**multivariate**	
	OR(CI 95%)	*p*	OR(CI 95%)	*p*
**Predictors**				
Age	1.01(0.97- 1.05)	0.58	**-**	**-**
Gender	0.82(0.34-1.99)	0.66	**-**	**-**
Hypertension	**0.32(0.12-0.84)**	**0.02**	**0.31(0.11- 0.87)**	**0.03**
Diabetes	1.18(0.47-2.94)	0.73	**-**	**-**
Dyslipidemia	0.53(0.22-1.29)	0.16	**-**	**-**
Cardiovascular Diseases	1.05(0.17-6.67)	0.96	**-**	**-**
Atrial Fibrillation	0.75(0.23-2.43)	0.63	**-**	**-**
Smoking History	1.06(0.35-3.05)	0.90	**-**	**-**
Toast Classification	1.01(0.85-1.43)	0.47	**-**	**-**
NIHSS	**1.14(1.06-1.24)**	**0.001**	**1.14(1.06-1.24)**	**0.001**
Ficolin-1	1.55(0.13-18.01)	0.73	**-**	**-**
Ficolin-3	0.95(0.88-1.03)	0.21	**-**	**-**

Exact *p* value for univariate and multivariate logistic regression analysis is reported. 95% Confidence Interval (CI 95%); Odds Ratio (OR). NIHSS: National Institutes of Health Stroke Scale; 3- month mRS: 3-month modified Rankin
