# Supplementary material for: Early ficolin-1 is a sensitive prognostic marker for functional outcome in ischemic stroke
Source: J Neuroinflammation. 2016 Jan 20;13:16. doi: 10.1186/s12974-016-0481-2 (PMC4721111; doi:10.1186/s12974-016-0481-2)
Supplement: Additional file 5: Table S5. — Leukocyte count in 48 h cohort. (PDF 64 kb) [file 12974_2016_481_MOESM5_ESM.pdf]

Table S5. Leukocyte count in 48h cohort

**Sperman rho**	correlation coefficient	*p*
**ficolin-1 vs inflammatory markers**		
Leukocyte Count (10^3/Ul)	0.34	0.008
Neutrophil Count (%)	0.33	0.08
Lymphocyte count (%)	- 0.36	0.003
N/L ratio	0.33	0.08

Spearman's rho for ficolin-1 vs leucocytes. N/L ratio (Neutrophil Lymphocyte Ratio).
